# Identification of chronic kidney disease patient characteristics influencing the renoprotective effects of febuxostat therapy: a retrospective follow-up study

**DOI:** 10.1186/s12882-017-0572-z

**Published:** 2017-05-18

**Authors:** Akinori Yamaguchi, Makoto Harada, Yosuke Yamada, Koji Hashimoto, Yuji Kamijo

**Affiliations:** Department of Nephrology, Shinshu University School of Medicine, 3-1-1 Asahi, Matsumoto, Nagano, 390-8621 Japan

**Keywords:** Chronic kidney disease, Febuxostat, Hyperuremia, Uric acid, Vascular risk factors

## Abstract

**Background:**

The ability of antihyperuricemic therapy to exert renoprotective effects in patients with chronic kidney disease (CKD) is controversial. In the present study, we studied patient characteristics that may mask favorable impact of antihyperuricemic therapy on the progression of CKD.

**Methods:**

This was a single-center, retrospective, follow-up study. One-hundred and seventy-eight CKD patients with hyperuricemia who received febuxostat therapy were included in this study. Mean serum uric acid (mUA) level after treatment and changes in estimated glomerular filtration rate (ΔeGFR) over 6 months were measured and their correlation was examined. Patients were divided into two groups based on mUA, and their ΔeGFR were compared. These analyses were evaluated in various subgroups.

**Results:**

Febuxostat therapy markedly decreased UA level in any CKD stage patients without resulting in serious adverse events. eGFRs of CKD patients in the mUA < 6.0 mg/dl group were maintained, whereas those in the mUA ≥ 6.0 mg/dl group decreased. A significant inverse correlation was observed between mUA and ΔeGFR (*r* = −0.16, *p* = 0.019). The renoprotective effects of febuxostat were significant in the following subgroups: male patients, age < 70 years, systolic blood pressure < 130 mmHg, normal cholesterol levels, and absence of diabetes. Coexisting vascular risk factors appear to exert additive masking effects against febuxostat renoprotection.

**Conclusions:**

The results of this study suggest that various vascular risk factors markedly attenuate the renoprotective effects of febuxostat.

**Electronic supplementary material:**

The online version of this article (doi:10.1186/s12882-017-0572-z) contains supplementary material, which is available to authorized users.

## Background

Hyperuricemia has been reported to be a risk factor for kidney dysfunction [1–5]. The mechanism underlying the progression of kidney dysfunction by activation of xanthine oxidase (XO) has been reported. Activation of XO increases reactive oxygen species (ROS) production and oxidative stress, as well as uric acid (UA) production, and causes vascular damage and organ dysfunction. Indeed, ROS is known to inactivate nitric oxide production and activates renin-angiotensin system, resulting in endothelial dysfunction and/or tubular injuries [6–8]. Alternatively, UA salts activate inflammasomes, triggering inflammation and leading to the development of tubular injuries [9–11]. Antihyperuricemic XO inhibitors, including febuxostat and allopurinol, are believed to suppress renal dysfunction via oxidative stress reduction and suppression of endothelial dysfunction and tubular injuries. However, some studies reported that hyperuricemia is not significantly related to the progression of kidney dysfunction in patients with advanced Chronic Kidney Disease (CKD) [12, 13], and that a renoprotective effect via lowering of the serum UA level was not observed in CKD patients [14]. Thus, the renoprotective effect of XO inhibitors is controversial, and further investigation is required.

We often find that CKD patients treated with antihyperuricemics show improved or maintained estimated glomerular filtration rate (eGFR) along with a reduction in serum UA level, whereas we also found cases showing none of the renoprotective effects. Since various risk factors for kidney dysfunction are involved in CKD progression, these factors could make it difficult to detect febuxostat-induced renoprotection.

In this study, we investigated whether antihyperuricemic therapy using febuxostat exerts renoprotective effects in CKD patients and which patient characteristics influence these renoprotective effects.

## Methods

### Study design and patients

This was a single-center, retrospective, observational study. Two-hundred thirty-two CKD patients underwent antihyperuricemic therapy using febuxostat at the Department of Nephrology in Shinshu University Hospital between May 2011 and October 2014. Patients were defined as having CKD if eGFR was <60 ml/min/1.73m^2^ and/or urine protein was >0.5 mg/day, or both urinary blood and protein were present over 3 months. We excluded patients with acute kidney injuries (AKI), acute nephritis, or rapidly progressive glomerulonephritis from the current study. Fifteen patients discontinued the febuxostat treatment owing to adverse events during the follow-up period, and 19 patients required renal replacement therapy. Follow-up clinical data in 20 patients could not be obtained owing to change in hospital or discontinuation of attendance. A total of 178 cases could be analyzed. No patients died during the follow-up period. Among these 178 patients, 118 did not previously use any antihyperuricemic agents and 60 were switched from allopurinol or benzbromarone to febuxostat. In Japan, a low initial dosage (10 mg/day) is recommended (maximum dose 60 mg/day). Moreover, febuxostat is known to show an increased AUC in the patients with severe kidney dysfunction (GFR < 30 ml/min) [15], and careful administration is necessary for these patients. For these reasons, febuxostat administration commenced at a dose of 10 mg/day in the antihyperuricemic-naïve group, and 10 or 20 mg/day in the group that was switched from other agents. The respective attending physicians determined dosage adjustments based on serum UA levels and its safety during the study period. All treatments patients received are considered standard care for their condition. Patient characteristics and clinical data were tabulated using medical record information. Changes in eGFR and serum UA levels at 6 months after febuxostat treatment initiation were analyzed statistically.

### Statistical analyses

To examine the effect of febuxostat therapy on changes in serum UA levels and eGFR, those parameters were measured at the time of febuxostat prescription, as well as 3 months and 6 months after therapy initiation. The correlation between mean serum uric acid level after treatment (mUA) and the change of eGFR after 6 months (6mΔeGFR) was analyzed using Spearman’s correlation. mUA was calculated as the mean serum UA level at 3 and 6 months. 6mΔeGFR was defined as the difference obtained by subtracting eGFR at treatment initiation from eGFR at 6 months. To investigate the effect of antihyperuricemic therapy for renal function lowering in CKD patients, we performed the correlation and ROC analyses using mUA and 6mΔeGFR data. Based on a calculated optimal cutoff level estimating progression of kidney dysfunction and standard goal of UA lowering therapy, the ΔeGFR was analyzed by dividing patients into mUA < 6.0 mg/dl and mUA ≥ 6.0 mg/dl groups.

The correlation between mUA and 6mΔeGFR was analyzed in all patients or in patient subgroups based on characteristics at the start of the therapy. Each patient’s clinical data were collected from their medical records. The patient characteristics examined were sex, age (< 70 vs. ≥ 70 years), systolic blood pressure (< 130 vs. ≥ 130 mmHg), the presence or absence of an abnormal cholesterol level, the presence or absence of diabetes mellitus, eGFR (< 30 vs. ≥ 30 ml/min/1.73 m^2^), urine protein level (< 0.5 vs. ≥ 0.5 g/gCre), and antihyperuricemic naive vs. agent switching group. The patients who were using a lipid-lowering drug or showed an abnormal level of LDL or HDL cholesterol (LDL cholesterol ≥140 mg/dl, HDL cholesterol <40 mg/dl) were defined as having symptoms of abnormal cholesterol levels. The patients who were using an antidiabetic or presented above 6.2% of HbA1c (NGSP) in a blood examination were defined as diabetes mellitus.

The subgroups, in which a significant correlation between mUA and 6mΔeGFR was detected, were then divided into the mUA < 6.0 mg/dl group and the mUA ≥ 6.0 mg/dl group to compare ΔeGFR. We also analyzed the other subgroups, which were separated based on numbers of risk factors.

Mann-Whitney U test was used to examine the statistical significance in continuous variables, and Fisher’s exact test was used to examine category variables. Wilcoxon signed-rank test was used to examine the statistical significance in time course changes in continuous variables. SPSS Statistics v.22.0 J (IBM Corp., Armonk, NY, USA) analysis software was used in this study. Differences were considered statistically significant at *p* < 0.05.

## Results

### Patient characteristics

Patient characteristics are presented in Table 1. Men accounted for 68% of the patient population, and the median age was 65 years. The median systolic and diastolic blood pressures at the start of febuxostat treatment were 130 mmHg (range: 85–180) and 76 mmHg (range: 45–110), respectively. Primary diseases of CKD included diabetic nephropathy in 19%, chronic nephritis in 20%, and nephrosclerosis in 19% of patients. The proportion of complications of diabetes mellitus, hypertension, or abnormal cholesterol levels was 25, 77, and 53% of patients, respectively. A history of coronary artery disease was confirmed in 7% of patients. The median eGFR was 27 ml/min/1.73 m^2^ (range: 7.3–101.7). Patient CKD stages at the start of treatment were 7% G1-2, 35% G3, and 58% G4-5. About 66% of patients had not used any antihyperuricemics previously, with the remaining 34% using allopurinol or benzbromarone. Febuxostat dose after 6 months was 10 mg/day in 58%, 20 mg/day in 34%, and 40 mg/day in 8% of patients.

**Table 1:** Patient characteristics

	All	sUA < 6.0 mg/dl	sUA ≥ 6.0 mg/dl	*p* value
Patients, n (%)	178	78	100	
Male, n (%)	121 (68%)	42 (54%)	79 (79%)	< 0.001
Age, years (range)	65 (15–89)	66 (17–89)	64 (15–88)	n.s.
Baseline systolic BP, mmHg (range)	130 (85–180)	129 (85–170)	130 (96–180)	n.s.
Baseline diastolic BP, mmHg (range)	76 (45–110)	74 (50–110)	76 (45–106)	n.s.
Primary diseases of CKD				
Diabetic nephropathy, n (%)	33 (19%)	9 (12%)	24 (24%)	n.s.
Chronic nephritis, n (%)	36 (20%)	19 (24%)	17 (17%)	n.s.
Nephrosclerosis, n (%)	34 (19%)	14 (18%)	20 (20%)	n.s.
Others, n (%)	75 (42%)	36 (46%)	39 (39%)	n.s.
Complications				
Diabetes mellitus, n (%)	45 (25%)	17 (22%)	28 (28%)	n.s.
Hypertension, n (%)	137 (77%)	56 (72%)	81 (81%)	n.s.
Abnormal cholesterol levels, n (%)	94 (53%)	34 (44%)	60 (60%)	n.s.
Coronary artery disease, n (%)	12 (7%)	4 (5%)	8 (8%)	n.s.
Estimated GFR at baseline, ml/min/1.73 m^2^	27.0 (7.3–101.7)	27.7 (7.3–78.9)	26.0 (9–101.7)	n.s.
Urine protein levels at baseline, g/gCre	0.67 (0.00–17.7)	0.45 (0.00–7.2)	0.78 (0.00–17.7)	n.s.
Serum uric acid at baseline, mg/dl	8.3 (3.9–12.6)	8.3 (3.9–10.7)	8.7 (5.2–12.6)	0.042
CKD stage				
G1–2, n (%)	13 (7%)	6 (8%)	7 (7%)	n.s.
G3, n (%)	62 (35%)	29 (37%)	33 (33%)	n.s.
G4–5, n (%)	103 (58%)	43 (55%)	60 (60%)	n.s.
Antihyperuricemics before febuxostat				
None, n (%)	118 (66%)	58 (74%)	60 (60%)	n.s.
Allopurinol, n (%)	44 (25%)	14 (18%)	30 (30%)	n.s
Benzbromarone, n (%)	14 (8%)	6 (8%)	8 (8%)	n.s.
Allopurinol and benzbromarone, n (%)	2 (1%)	0 (0%)	2 (2%)	n.s.
Febuxostat dose after 6 months				
10 mg/day, n (%)	103 (58%)	51 (66%)	52 (52%)	n.s.
20 mg/day, n (%)	61 (34%)	22 (28%)	40 (39%)	n.s.
40 mg/day, n (%)	14 (8%)	5 (6%)	9 (9%)	n.s.

### Adverse events

There were 15 cases wherein febuxostat treatment was discontinued during the follow-up period owing to adverse events (6.5% of 232 cases, Additional file 1: Table S1). No serious adverse events developed in any of these cases, and discontinuation of the drug led to rapid disappearance of the adverse reaction in all cases. Compared to the group of patients who could continue the febuxostat treatment, the adverse event group included a significantly higher percentage of female patients (67% of 15 cases). In most patients (93% of 15 cases), the adverse events developed during the initial dosing of febuxostat at 10 mg/day.

### Antihyperuricemic effect of febuxostat

**Fig. 1:**
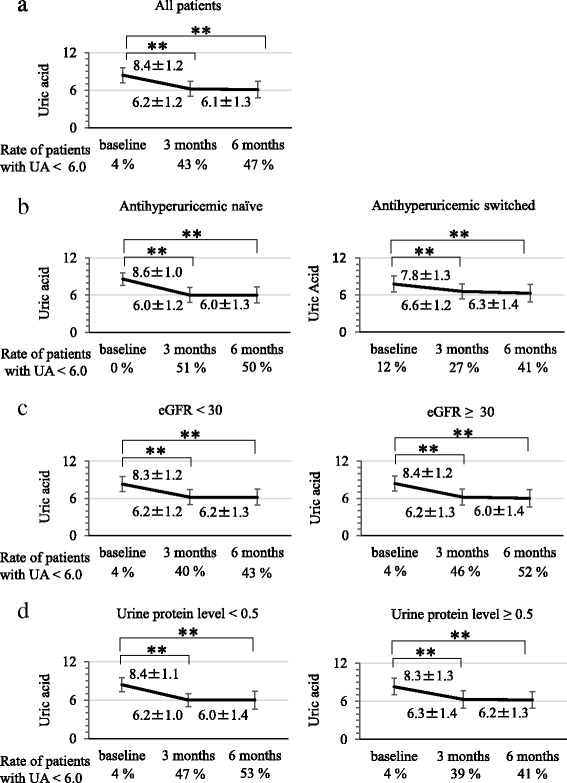
Alteration of serum uric acid level due to febuxostat therapy. The changes in serum uric acid (UA) levels after febuxostat administration (baseline, 3 and 6 months after treatment initiation). **a** All patients (*n* = 178), **b** antihyperuricemic naïve (*n* = 118) vs. antihyperuricemic switched groups (*n* = 60), **c** eGFR <30 (*n* = 75) vs. eGFR ≥30 ml/min/1.73 m^2^ groups (*n* = 103), **d** urine protein level < 0.5 (*n* = 81) vs. urine protein level ≥ 0.5 g/gCre groups (*n* = 92). The UA levels represent the mean ± SD. **p < 0.01, compared with baseline UA level in each group (Wilcoxon signed-rank test)

The changes in serum UA levels after febuxostat administration are shown in Fig. 1. The all patients, antihyperuricemic naive, and antihyperuricemic switched groups all showed significant decreases of serum UA levels at 3 and 6 months after treatment initiation. The mUA of all patients was 6.2 ± 1.2 mg/dl. Dividing the patients into subgroups based on their eGFR or urine protein level revealed that febuxostat exerted a significant antihyperuricemic effect, which were identical among the groups. Six months after therapy, the serum UA level was lower than 6.0 mg/dl in 40–50% of patients in all groups.

### eGFR changes due to febuxostat therapy

The scatter plot and receiver operating characteristic (ROC) curve using mUA and 6mΔeGFR data are shown in Additional file 2: Figure S1. A significant inverse correlation was detected between mUA and 6mΔeGFR in all patients, suggesting that the decrease of mUA leads to attenuation of CKD-induced eGFR decline. ROC analysis revealed that the calculated optimal cutoff level of mUA estimating progression of kidney dysfunction was 6.25 mg/dl (AUC 0.573, sensitivity 59.4%, specificity 61.0%). Traditionally, the standard goal of antihyperuricemic therapy has been set at mUA < 6.0 mg/dl in most of the clinical studies, including a report assessing renoprotective effect of antihyperuricemics [16]. Since this traditional target UA level is very close to our calculated cutoff level (mUA < 6.25 mg/dl), mUA < 6.0 mg/dl (sensitivity 67.3%, specificity 52.0%) was used as the cutoff level to be consistent with the past studies.

**Fig. 2:**
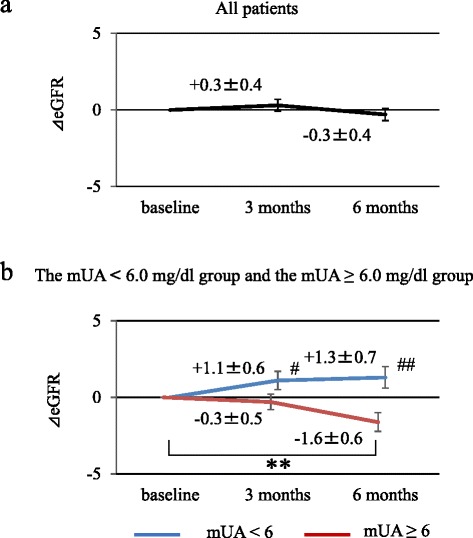
Alteration of ΔeGFR due to febuxostat therapy. The changes of eGFR (ΔeGFR) after febuxostat administration (baseline, 3 and 6 months after treatment initiation). **a** All patients (*n* = 178). **b** The ΔeGFR was analyzed by dividing patients into mean serum uric acid level (mUA) < 6.0 mg/dl (blue line, *n* = 78) vs. mUA ≥ 6.0 mg/dl (red line, *n* = 100) groups. ΔeGFR represent the mean ± SE. ΔeGFR was defined as the difference obtained by subtracting baseline eGFR from post-treatment eGFR. **p < 0.01; compared with baseline UA level in each group (Wilcoxon signed-rank test). # *p* < 0.05, ## *p* < 0.01; comparison between mUA < 6.0 and mUA ≥ 6.0 mg/dl groups (Mann-Whitney U test)

ΔeGFR measurements after febuxostat administration are shown in Fig. 2. No significant change was seen in ΔeGFR at 3 and 6 months after treatment initiation when all patients were grouped together. However, dividing the patients into mUA < 6.0 mg/dl and mUA ≥ 6.0 mg/dl groups led to the detection of a significant eGFR decrease in the mUA ≥ 6.0 mg/dl group. No significant change in eGFR was noted in the mUA < 6.0 mg/dl group. Comparing the two groups, eGFR was significantly higher at 3 and 6 months in the mUA < 6.0 mg/dl group than in the mUA ≥ 6.0 mg/dl group, indicating the renoprotective effects associated with lowering of serum UA levels.

### Relationship between serum UA level and eGFR change in various subgroups

The relationship between mUA and 6mΔeGFR showed a wide variation when all patients were grouped together, resulting in a low correlation coefficient. Therefore, the correlation was examined relative to specific patient characteristics after dividing the patients into various subgroups (Additional file 3: Figure S2). A significant inverse correlation between mUA and 6mΔeGFR was detected in the following subgroups: male patients, patient age < 70 years, systolic blood pressure < 130 mmHg, absence of abnormal cholesterol levels, and absence of diabetes mellitus. On the contrary, no inverse correlation was observed in the opposite subgroups: female patients, patient age ≥ 70 years, systolic blood pressure ≥ 130 mmHg, abnormal cholesterol levels, and diabetes mellitus. These analyses indicate that effects of febuxostat therapy can be affected by various vascular risk factors. Additionally, no significant inverse correlation between mUA and 6mΔeGFR was detected in subgroups divided based on eGFR 30 ml/min/1.73 m^2^, urine protein levels 0.5 g/gCre, or antihyperuricemic naïve and agent switching groups (data not shown).

**Fig. 3:**
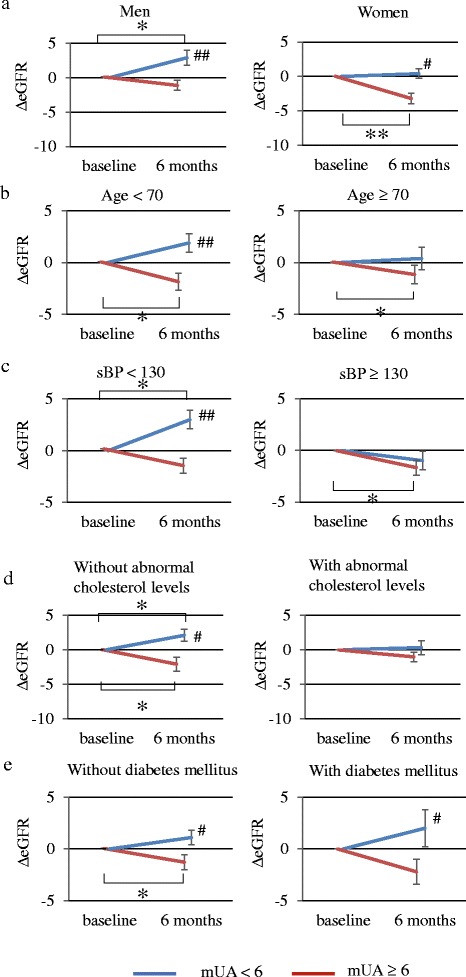
Difference in 6mΔeGFR in mUA < 6.0 vs. ≥ 6.0 groups in various subgroups. ΔeGFR after 6 months (6mΔeGFR) were analyzed by dividing patients into mean serum uric acid (mUA) level < 6.0 mg/dl vs. mUA ≥ 6.0 mg/dl groups. The analyses were performed in various subgroups. **a** Men (*n* = 121) vs. Women (*n* = 57), **b** Age < 70 (*n* = 107) vs. age ≥ 70 years (*n* = 71), **c** systolic blood pressure (sBP) < 130 (*n* = 95) vs. sBP ≥ 130 mmHg (*n* = 83), **d** normal cholesterol levels (*n* = 84) vs. abnormal cholesterol levels (*n* = 94), **e** absence of diabetes mellitus (*n* = 133) vs. diabetes mellitus (*n* = 45). 6mΔeGFR represent the mean ± SE. *p < 0.05, **p < 0.01, compared with baseline UA level in each group (Wilcoxon signed-rank test). *# p* < 0.05, *## p* < 0.01; comparison between mUA < 6.0 and mUA ≥ 6.0 mg/dl groups (Mann-Whitney U test)

The subgroups showing an inverse correlation between mUA and 6mΔeGFR were further divided based on the mUA level to compare their 6mΔeGFR (Figure 3). In the subgroups of men, aged <70 years, systolic blood pressure < 130 mmHg, normal cholesterol levels, and absence of diabetes, a significant increase of eGFR was detected in the mUA < 6.0 mg/dl group compared to that in the mUA ≥ 6.0 mg/dl group. In these subgroups, a significant difference in 6mΔeGFR was detected between the mUA < 6.0 and mUA ≥ 6.0 mg/dl groups. On the contrary, the opposite subgroups including women, aged ≥70 years, systolic blood pressure ≥ 130 mmHg, abnormal cholesterol levels, and diabetes, exhibited slight changes in eGFR. In these subgroups with vascular risk factors, a significant difference in 6mΔeGFR was obscure between mUA < 6.0 and mUA ≥ 6.0 mg/dl groups.

### Relationship between mUA level and eGFR change in subgroups divided based on the number of vascular risk factors

The correlations between mUA and 6mΔeGFR in subgroups divided based on the number of these vascular risk factors are shown in Additional file 4: Figure S3. In the subgroup without vascular risk factors, there is a strong significant inverse correlation between mUA and 6mΔeGFR (*r* = −0.4, *p* = 0.029, *n* = 30). In the subgroups with one to two vascular risk factors, a significant inverse correlation between mUA and 6mΔeGFR became obscure, and the correlation completely vanished in the subgroup with more than three cardiovascular risk factors.

**Fig. 4:**
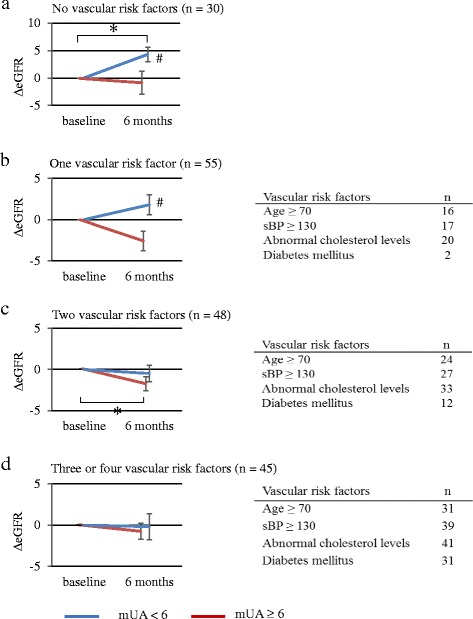
Difference in 6mΔeGFR in subgroups divided based on the number of vascular risk factors. ΔeGFR after 6 months (6mΔeGFR) were analyzed by dividing patients into mean serum uric acid (mUA) level < 6.0 mg/dl vs. mUA ≥ 6.0 mg/dl groups. The analyses were performed in subgroups divided based on the number of vascular risk factors. **a** No vascular risk factors (*n* = 30), **b** with one vascular risk factors (*n* = 55), **c** with two vascular risk factors (*n* = 48), **d** with three or four vascular risk factors (*n* = 45). ΔeGFR represent the mean ± SE. *p < 0.05, compared with baseline UA level in each group (Wilcoxon signed-rank test). *# p* < 0.05; comparison between mUA < 6.0 and mUA ≥ 6.0 mg/dl groups (Mann-Whitney U test)

The changes of eGFR in each subgroup, which were further divided based on mUA levels, are shown in Fig. 4. In the subgroups with one or less risk factors, eGFR increased in mUA < 6.0 groups, and significant differences in 6mΔeGFR were detected between mUA < 6.0 group and mUA ≥ 6.0 group. In the subgroups with more two risk factors, improvement of eGFR via hyperuricemic therapy, as well as significant differences in 6mΔeGFR between mUA < 6.0 group and mUA ≥ 6.0 group, were not detected.

## Discussion

Attempts to control serum UA levels using previous antihyperuricemic medicines, including benzbromarone and allopurinol, in patients with kidney dysfunction, have proved difficult. Since benzbromarone is an agent that enhances urinary excretion of UA, its antihyperuricemic effect is attenuated by kidney dysfunction. Another XO inhibitor, allopurinol, is mainly excreted by urine; therefore, its serum concentration and adverse events are increased in the patients with kidney dysfunction, requiring dose reduction from an early phase of CKD and resulting in poor control of serum UA levels. Since febuxostat is excreted via dual excretion pathways, urine 49.1% and fecal 44.9% [17], febuxostat can exert strong antihyperuricemic effect without serious adverse events even for patients with kidney dysfunction without changing dosage [18, 19]. Although careful febuxostat administration is necessary for patients with severe kidney dysfunction (GFR < 30 ml/min), its metabolic characteristics are advantageous compared to those of allopurinol. Indeed, this study demonstrates that febuxostat therapy is effective in controlling serum UA levels in patients at any CKD stage. The percentage of patients for whom febuxostat could be administered continuously for 6 months was high, as the incidence of serious adverse events was very low. In the very small number of CKD patients who developed an adverse event, the symptoms improved rapidly after drug discontinuation. These results are consistent with reports stating that febuxostat treatment exerts a more potent antihyperuricemic effect with an equivalent incidence of adverse events compared to allopurinol in patients with kidney dysfunction [18, 19]. Therefore, these results indicate that febuxostat treatment is useful for lowering serum UA levels in CKD patients.

Many studies have reported that hyperuricemia is a risk factor for the progression of kidney dysfunction in the general population, diabetes mellitus, AKI and CKD patients [1–5]. Therefore, antihyperuricemic therapy is expected to contribute to kidney protection. In this study, we showed that the decline of eGFR was remarkable in CKD patients with poor control of UA level (mUA ≥ 6.0 mg/dl), whereas it was likely to be ameliorated by febuxostat therapy in patients with good control of UA level (mUA < 6.0 mg/dl). In addition, we detected an inverse correlation between mUA and 6mΔeGFR, suggesting that further decreases in mUA after febuxostat administration contribute to suppression of CKD-induced eGFR decline. These findings support the hypothesis that lowering serum UA level is renoprotective. These results are consistent with previous studies reporting that antihyperuricemic therapy in patients with CKD ameliorates eGFR reduction [20, 21]. However, the renoprotective effects of antihyperuricemic therapy in CKD patients are controversial in several small-scale randomized controlled trials (RCTs) and various follow-up studies involving CKD patients. Two placebo-controlled small-scale RCTs in which allopurinol or febuxostat were used reported that CKD-induced eGFR decline was suppressed more in the antihyperuricemic group than in the placebo group [20, 21]. Levy DG et al. performed antihyperuricemic therapy for 3 years using allopurinol, febuxostat, or probenecid for patients with hyperuricemia, and found that renal dysfunction could be suppressed in patients with controlled UA levels at <6 mg/dL [16]. In our study, we also found that deterioration of kidney function could be suppressed in patients with UA levels controlled at lower than 6 mg/dl, supporting the above reports. In contrast, a RCT involving 40 patients with IgA nephropathy and CKD stage 1–3 reported no renoprotective effects of a 6-month allopurinol therapy course [14]. The lack of consensus among many clinical studies regarding a renoprotective effect by antihyperuricemic therapy may be due to the differences of patient characteristics in each study.

The current subgroup analyses demonstrated that patients with parameters including male, < 70 years, systolic blood pressure < 130 mmHg, no abnormal cholesterol levels, or no diabetes mellitus are more likely to show an inverse correlation between mUA level and 6mΔeGFR, as well as amelioration of eGFR decline by UA lowering, than their opposite subgroups. This result suggests that the renoprotective effects of febuxostat differ among CKD patients. The factors masking the renoprotective effects detected in this study, including aging, hypertension, hyperlipidemia, and diabetes mellitus, are important vascular risk factors causing endothelial dysfunction and arteriosclerosis. XO activation and hyperuricemia are also thought to be vascular injury factors. XO activation increases oxidative stress, suppresses nitric oxide production, and activates the renin-angiotensin system [6–8]. Hyperuricemia is reported to induce arteriolopathy of preglomerular vessels, which impairs endothelial function and the autoregulatory response of afferent arterioles, resulting in glomerular hypertension [8]. Antihyperuricemic therapy using febuxostat is expected to improve these vascular events induced by XO activation and hyperuricemia; however, its vascular protective effects may be masked by other vascular risk factors. The current results demonstrate that eGFR is likely to decrease despite optimal control of the UA level in CKD patients with various vascular risk factors, and that coexisting vascular risk factors exert additive masking effects against febuxostat renoprotection. To detect the reliable renoprotective effects of antihyperuricemic therapy, clinical studies in which control and interventional groups have small number of background vascular risk factors will be needed in the future.

The current findings do not negate the therapeutic significance of febuxostat treatment for CKD patients with various risk factors such as diabetes mellitus. Zoppini et al. reported that hyperuricemia is an independent risk factor for CKD progression in patients with type 2 diabetes [2]. We believe that antihyperuricemic therapy is an important interventional strategy to protect kidney function even in patients with diabetes mellitus, because it is expected to ameliorate additional vascular damage induced by hyperuricemia.

Limitations of this study include a small number of subjects from a short duration, single-center retrospective follow-up study. The analyses with small n-number and short-duration follow-up might show borderline significance values, especially in subgroup analyses. However, we believe that the findings of the current study would provide useful information for clinical studies in the future, by identifying the patient characteristics influencing the renoprotective effects of febuxostat therapy. Further clarification of the differences in the renoprotection effects by antihyperuricemic therapy among various patient subgroups will require future examination in large-scale, extended-duration, multi-center prospective studies. Second, the dosage of febuxostat in Japan differs from that in other countries due to safety assessment and the dose ranging studies. The proportions of patients with serum UA < 6.0 mg/dl were 40 mg, 56%; 80 mg, 76%; and 120 mg, 94% in the dose ranging study in USA [22], whereas they were 20 mg, 46.5%; 40 mg, 82.9%; 60 mg, 83.3%; and 80 mg, 87.8% in Japan [23]. These findings suggest that low-dose febuxostat therapy can lower serum UA levels in Japanese patients to a greater extent than in European and Americans patients. From the results of the APEX, FACT, and CONFIRMS studies [17, 18, 24], febuxostat dose was set at 40–80 mg/day in USA and 80–120 mg/day in Europe, whereas the initial dose was set at 10 mg/day, with careful gradual dose increase of febuxostat (maximum 60 mg/day), in Japan based on the results of the allopurinol-controlled comparative studies and a long-term study [25–27]. This suggested a possible difference in antihyperuricemic effect due to factors such as genetic differences, body mass index, or dietary habits. Therefore, the result of the present study was not directly applicable to patients of other countries. Third, the current study included only a small of number female patients, in whom a high number of adverse events were reported, making the evaluation of the clinical effects of febuxostat therapy in female patients inconclusive. Therefore further research for female patients is necessary. In addition, factors not examined in this study might have influenced the study results; therefore, careful interpretation of the results would be required.

## Conclusions

Febuxostat therapy was effective in controlling the UA level in patients at any CKD stage and was highly safe. In the well-controlled UA level (mUA < 6.0 mg/dl) group, antihyperuricemic therapy using febuxostat may exert renoprotective effects. The effects are more likely to be detected in patients free of vascular risk factors, such as aging, hypertension, hyperlipidemia, and diabetes mellitus.

## Additional files

Additional file 1: Table S1. Adverse events. There were 15 cases wherein febuxostat treatment was discontinued owing to various adverse events. In most patients, the adverse events developed during the initial dosing of febuxostat at 10 mg/day. (DOCX 18 kb)

Additional file 2: Figure S1. Correlation between mUA and 6mΔeGFR, and ROC curve indicating mUA cutoff level estimating CKD progression. (a) Scatter plot indicating the correlation between mean serum uric acid (mUA) level and ΔeGFR after 6 months (6mΔeGFR) (*n* = 178). A correlation coefficient (r) and *p*-value (p) were analyzed using Spearman’s correlation analysis. (b) ROC analysis revealed that the calculated optimal cutoff level of mUA estimating progression of kidney dysfunction was 6.25 mg/dl (AUC 0.573, sensitivity 59.4%, specificity 61.0%). (PPTX 83 kb)

Additional file 3: Figure S2. Correlation between mUA and 6mΔeGFR in various subgroups. Scatter plots indicating the correlation between mean serum uric acid (mUA) level and ΔeGFR after 6 months (6mΔeGFR) in various subgroups. (a) Men (*n* = 121) vs. Women (*n* = 57), (b) Age < 70 (*n* = 107) vs. age ≥ 70 years (*n* = 71), (c) systolic blood pressure (sBP) < 130 (*n* = 95) vs. sBP ≥ 130 mmHg (*n* = 83), (d) normal cholesterol levels (*n* = 84) vs. abnormal cholesterol levels (*n* = 94), (e) absence of diabetes mellitus (*n* = 133) vs. diabetes mellitus (*n* = 45). A correlation coefficient (r) and *p*-value (p) were analyzed using Spearman’s correlation analysis. (PPTX 151 kb)

Additional file 4: Figure S3. Correlation between mUA and 6mΔeGFR in subgroups divided based on the number of vascular risk factors. Scatter plots indicating the correlation between mean serum uric acid (mUA) level and ΔeGFR after 6 months (6mΔeGFR) in subgroups divided based on the number of vascular risk factors. (a) No vascular risk factors (*n* = 30), (b) one vascular risk factor (*n* = 55), (c) two vascular risk factors (*n* = 48), (d) three or four vascular risk factors (*n* = 45). A correlation coefficient (r) and *p*-value (p) were analyzed using Spearman’s correlation analysis. (PPTX 99 kb)

## Abbreviations

6mΔeGFR: The change in eGFR after 6 months
AKI: Acute kidney injury
CKD: Chronic kidney diseases
eGFR: Estimated glomerular filtration rate
mUA: Mean uric acid
RCT: Randomized controlled trials
ROC: Receiver operating characteristic
ROS: Reactive oxygen species
UA: Uric acids
XO: Xanthine oxidase
ΔeGFR: The change in eGFR
