# Supplementary material for: Identification of chronic kidney disease patient characteristics influencing the renoprotective effects of febuxostat therapy: a retrospective follow-up study
Source: BMC Nephrol. 2017 May 18;18:162. doi: 10.1186/s12882-017-0572-z (PMC5437587; doi:10.1186/s12882-017-0572-z)
Supplement: Supplementary file 1 — Adverse events. There were 15 cases wherein febuxostat treatment was discontinued owing to various adverse events. In most patients, the adverse events developed during the initial dosing of febuxostat at 10 mg/day. (DOCX 18 kb) [file 12882_2017_572_MOESM1_ESM.docx]

TableS1|Adverse events

| Case | Age ranges | eGFR  (ml/min/1.73 m^2^) | Dose  (mg/day) | Adverse events |
| --- | --- | --- | --- | --- |
| 1 | 70s | 27 | 10 | Rash |
| 2 | 80s | 9 | 10 | Rash |
| 3 | 70s | 22 | 10 | Rash |
| 4 | 70s | 21 | 10 | Liver transaminase abnormality |
| 5 | 60s | 22 | 10 | Liver transaminase abnormality |
| 6 | 60s | 29 | 10 | Overlowering of uric acid |
| 7 | 20s | 87 | 10 | Overlowering of uric acid |
| 8 | 30s | 11 | 10 | Malaise |
| 9 | 80s | 9 | 10 | Malaise |
| 10 | 50s | 49 | 10 | Edema |
| 11 | 80s | 46 | 10 | Fatigability |
| 12 | 70s | 42 | 10 | Anorexia |
| 13 | 50s | 26 | 10 | Antipruritic |
| 14 | 50s | 7 | 10 | Tubular injury |
| 15 | 50s | 8 | 20 | Diarrhea |
