# Supplementary material for: Identification of chronic kidney disease patient characteristics influencing the renoprotective effects of febuxostat therapy: a retrospective follow-up study
Source: BMC Nephrol. 2017 May 18;18:162. doi: 10.1186/s12882-017-0572-z (PMC5437587; doi:10.1186/s12882-017-0572-z)
Supplement: Supplementary file 2 — Correlation between mUA and 6mΔeGFR, and ROC curve indicating mUA cutoff level estimating CKD progression. (a) Scatter plot indicating the correlation between mean serum uric acid (mUA) level and ΔeGFR after 6 months (6mΔeGFR) (n = 178). A correlation coefficient (r) and p-value (p) were analyzed using Spearman’s correlation analysis. (b) ROC analysis revealed that the calculated optimal cutoff level of mUA estimating progression of kidney dysfunction was 6.25 mg/dl (AUC 0.573, sensitivity 59.4%, specificity 61.0%). (PPTX 83 kb) [file 12882_2017_572_MOESM2_ESM.pptx]

## Slide 1
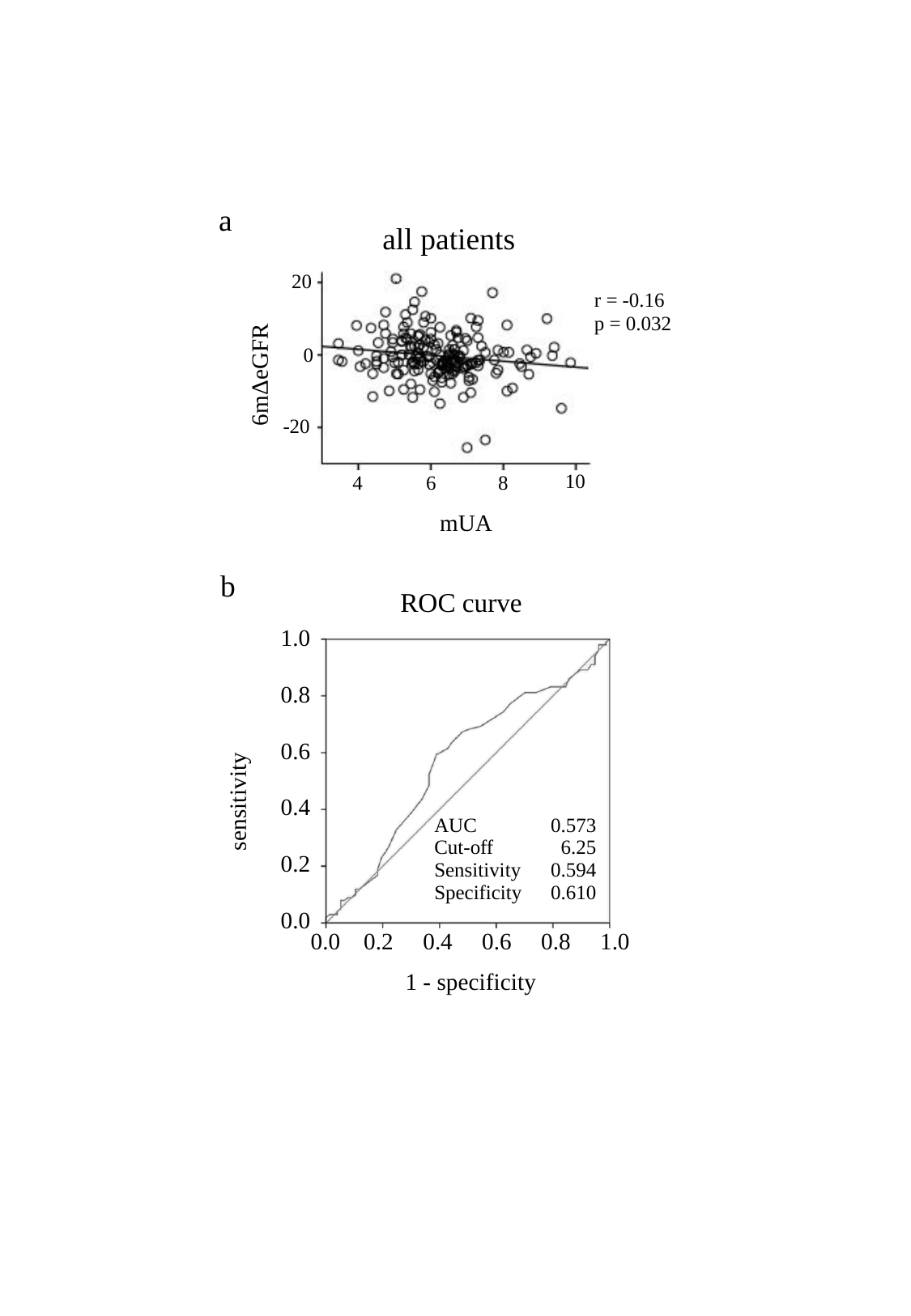

a
all patients
20
r = -0.16
p = 0.032
6mΔeGFR
0
-20
10
4
6
8
mUA
b
ROC curve
1.0
0.8
0.6
0.4
0.2
0.0
sensitivity
| AUC Cut-off Sensitivity Specificity | 0.573 6.25 0.594 0.610 |
| --- | --- |
0.0 0.2 0.4 0.6 0.8 1.0
1 - specificity
