# Supplementary material for: Identification of chronic kidney disease patient characteristics influencing the renoprotective effects of febuxostat therapy: a retrospective follow-up study
Source: BMC Nephrol. 2017 May 18;18:162. doi: 10.1186/s12882-017-0572-z (PMC5437587; doi:10.1186/s12882-017-0572-z)
Supplement: Supplementary file 3 — Correlation between mUA and 6mΔeGFR in various subgroups. Scatter plots indicating the correlation between mean serum uric acid (mUA) level and ΔeGFR after 6 months (6mΔeGFR) in various subgroups. (a) Men (n = 121) vs. Women (n = 57), (b) Age < 70 (n = 107) vs. age ≥ 70 years (n = 71), (c) systolic blood pressure (sBP) < 130 (n = 95) vs. sBP ≥ 130 mmHg (n = 83), (d) normal cholesterol levels (n = 84) vs. abnormal cholesterol levels (n = 94), (e) absence of diabetes mellitus (n = 133) vs. diabetes mellitus (n = 45). A correlation coefficient (r) and p-value (p) were analyzed using Spearman’s correlation analysis. (PPTX 151 kb) [file 12882_2017_572_MOESM3_ESM.pptx]

## Slide 1
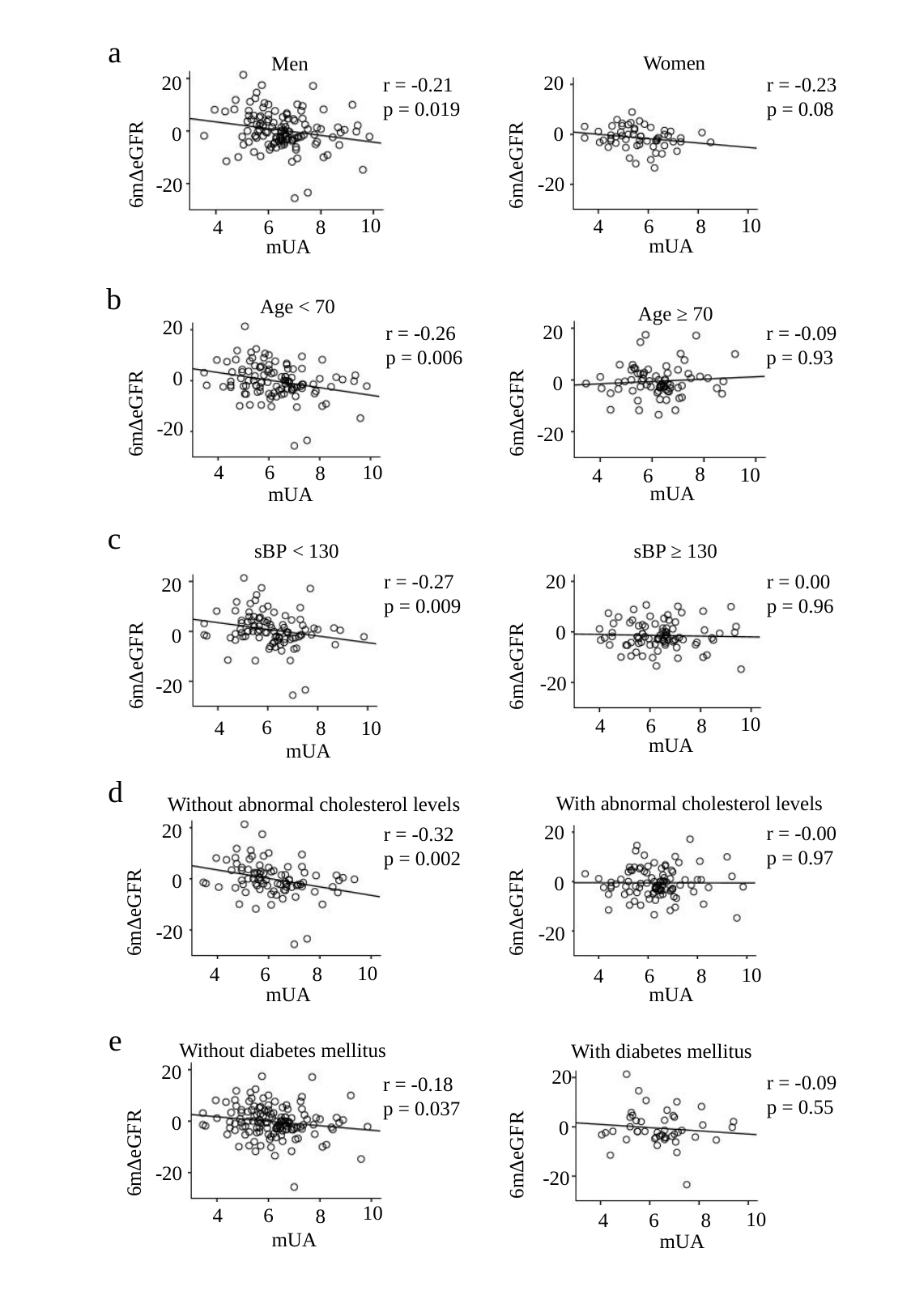

a
Women
Men
20
20
r = -0.21
p = 0.019
r = -0.23
p = 0.08
0
0
6mΔeGFR
6mΔeGFR
-20
-20
10
10
4
6
8
4
6
8
mUA
mUA
b
Age < 70
Age ≥ 70
20
20
r = -0.26
p = 0.006
r = -0.09
p = 0.93
0
6mΔeGFR
0
6mΔeGFR
-20
-20
10
4
6
8
8
10
4
6
mUA
mUA
c
sBP < 130
sBP ≥ 130
20
r = 0.00
p = 0.96
r = -0.27
p = 0.009
20
0
0
6mΔeGFR
6mΔeGFR
-20
-20
10
4
6
8
6
4
8
10
mUA
mUA
d
With abnormal cholesterol levels
Without abnormal cholesterol levels
20
20
r = -0.00
p = 0.97
r = -0.32
p = 0.002
0
0
6mΔeGFR
6mΔeGFR
-20
-20
10
4
6
8
10
4
6
8
mUA
mUA
e
Without diabetes mellitus
With diabetes mellitus
20
20
r = -0.09
p = 0.55
r = -0.18
p = 0.037
0
6mΔeGFR
0
6mΔeGFR
-20
-20
10
4
6
8
10
4
6
8
mUA
mUA
