# Supplementary material for: Identification of chronic kidney disease patient characteristics influencing the renoprotective effects of febuxostat therapy: a retrospective follow-up study
Source: BMC Nephrol. 2017 May 18;18:162. doi: 10.1186/s12882-017-0572-z (PMC5437587; doi:10.1186/s12882-017-0572-z)
Supplement: Supplementary file 4 — Correlation between mUA and 6mΔeGFR in subgroups divided based on the number of vascular risk factors. Scatter plots indicating the correlation between mean serum uric acid (mUA) level and ΔeGFR after 6 months (6mΔeGFR) in subgroups divided based on the number of vascular risk factors. (a) No vascular risk factors (n = 30), (b) one vascular risk factor (n = 55), (c) two vascular risk factors (n = 48), (d) three or four vascular risk factors (n = 45). A correlation coefficient (r) and p-value (p) were analyzed using Spearman’s correlation analysis. (PPTX 99 kb) [file 12882_2017_572_MOESM4_ESM.pptx]

## Slide 1
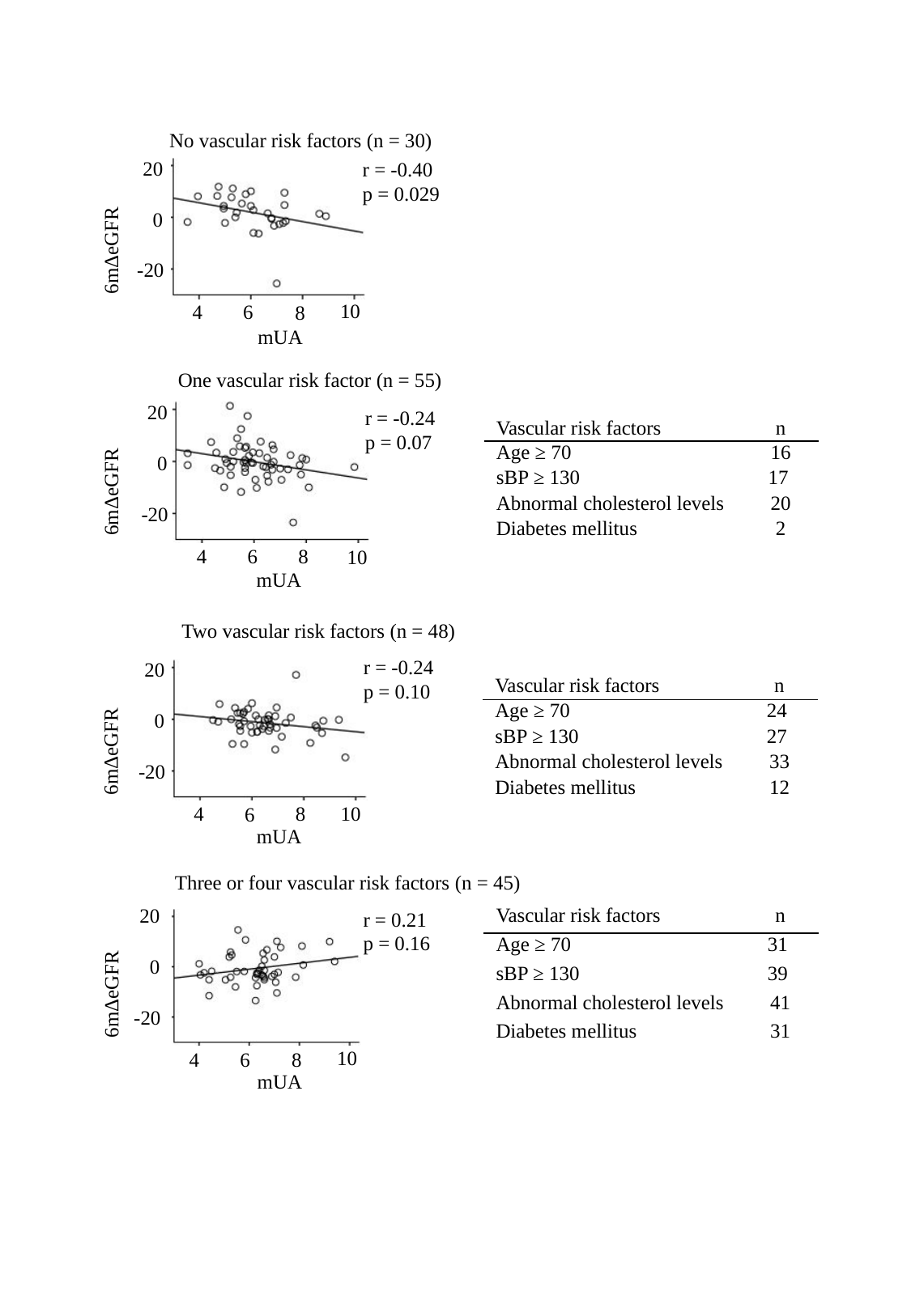

No vascular risk factors (n = 30)
20
r = -0.40
p = 0.029
0
6mΔeGFR
-20
10
4
6
8
mUA
One vascular risk factor (n = 55)
20
r = -0.24
p = 0.07
| Vascular risk factors | n |
| --- | --- |
| Age ≥ 70 | 16 |
| sBP ≥ 130 | 17 |
| Abnormal cholesterol levels | 20 |
| Diabetes mellitus | 2 |
0
6mΔeGFR
-20
4
6
8
10
mUA
Two vascular risk factors (n = 48)
r = -0.24
p = 0.10
20
| Vascular risk factors | n |
| --- | --- |
| Age ≥ 70 | 24 |
| sBP ≥ 130 | 27 |
| Abnormal cholesterol levels | 33 |
| Diabetes mellitus | 12 |
0
6mΔeGFR
-20
4
8
10
6
mUA
Three or four vascular risk factors (n = 45)
20
r = 0.21
p = 0.16
| Vascular risk factors | n |
| --- | --- |
| Age ≥ 70 | 31 |
| sBP ≥ 130 | 39 |
| Abnormal cholesterol levels | 41 |
| Diabetes mellitus | 31 |
0
6mΔeGFR
-20
10
4
6
8
mUA
